# Genomic variability in Zika virus in GBS cases in Colombia

**DOI:** 10.1371/journal.pone.0313545

**Published:** 2024-11-19

**Authors:** Nelson Rivera-Franco, Diana López-Alvarez, Andrés Castillo, Erica Aristizabal, Daniela Puiu, Steven L. Salzberg, Carlos A. Pardo, Beatriz Parra, on behalf of NEAS

**Affiliations:** 1 Laboratorio de Técnicas y Análisis Ómicos—TAOLab/CiBioFi, Facultad de Ciencias Naturales y Exactas, Universidad del Valle, Cali, Valle del Cauca, Colombia; 2 Grupo VIREM—Virus Emergentes y Enfermedad, Escuela de Ciencias Básicas, Facultad de Salud, Universidad del Valle, Cali, Valle del Cauca, Colombia; 3 Department of Neurology & Pathology, Johns Hopkins University School of Medicine, Baltimore, Maryland, United States of America; 4 Departamento de Ciencias Biológicas, Facultad de Ciencias Agropecuarias, Universidad Nacional de Colombia, Palmira, Valle del Cauca, Colombia; 5 Center for Computational Biology, Johns Hopkins University, Baltimore, Maryland, United States of America; 6 Department of Biomedical Engineering, Johns Hopkins University, Baltimore, Maryland, United States of America; Universidad Cooperativa de Colombia, COLOMBIA

## Abstract

Major clusters of Guillain-Barré Syndrome (GBS) emerged during the Zika virus (ZIKV) outbreaks in the South Pacific and the Americas from 2014 to 2016. The factors contributing to GBS susceptibility in ZIKV infection remain unclear, although considerations of viral variation, patient susceptibility, environmental influences, and other potential factors have been hypothesized. Studying the role of viral genetic factors has been challenging due to the low viral load and rapid viral clearance from the blood after the onset of Zika symptoms. The prolonged excretion of ZIKV in urine by the time of GBS onset, when the virus is no longer present in the blood, provides an opportunity to unravel whether specific ZIKV mutations are related to the development of GBS in certain individuals. This study aimed to investigate the association between specific ZIKV genotypes and the development of GBS, taking advantage of a unique collection of ZIKV-positive urine samples obtained from GBS cases and controls during the 2016 ZIKV outbreak in Colombia. Utilizing Oxford-Nanopore technology, we conducted complete genome sequencing of ZIKV in biological samples from 15 patients with GBS associated with ZIKV and 17 with ZIKV infection without neurological complications. ZIKV genotypes in Colombia exhibited distribution across three clades (average bootstrap of 90.9±14.9%), with two clades dominating the landscape. A comparative analysis of ZIKV genomes from GBS and non-neurological complications, alongside 1368 previously reported genomes, revealed no significant distinctions between the two groups. Both genotypes were similarly distributed among observed clades in Colombia. Furthermore, no variations were identified in the amino acid composition of the viral genome between the two groups. Our findings suggest that GBS in ZIKV infection is perhaps associated with patient susceptibility and/or other para- or post-infectious immune-mediated mechanisms rather than with specific ZIKV genome variations.

## Introduction

The resurgence of the Zika virus (ZIKV) in 2014 in the South Pacific and its subsequent spread in the Americas from 2015 to 2016, with the simultaneous surge in cases of the Guillain-Barré Syndrome (GBS), brought attention to the correlation between ZIKV and this usually rare neurological condition [1,2]. GBS is an acute neuroinflammatory disorder affecting the peripheral nervous system, often associated with viral and bacterial infections [3]. Despite the shift from the epidemic to the endemic phase of ZIKV infection in 2017 in numerous countries, pockets of ZIKV infection persist, particularly in the Americas and other regions [4–6] where mosquito-borne transmission, facilitated by *Aedes* genus mosquitoes (primarily *Aedes aegypti* and *Aedes albopictus*), remains a concern [7,8].

Beyond the typical clinical manifestations of ZIKV infection, including fever, rash, myalgia, arthralgia, and conjunctivitis, a minority of patients encounter acute neurological complications like GBS, encephalitis, and myelitis [2,9–11]. Although demonstrating the direct causative role of ZIKV in GBS proves challenging, a clear association between ZIKV infection and an elevated GBS risk exists. The initial link between GBS and ZIKV infection emerged through the temporal correlation observed during the outbreak of ZIKV in French Polynesia in 2014 [2]. This connection was subsequently reinforced by a similar upsurge in GBS incidence during the ZIKV outbreak in the Americas from 2015 to 2016 [1,9,12]. GBS is estimated to occur in approximately 2 cases per 10,000 ZIKV infections [13]. A significant gap in our understanding of the mechanisms connecting ZIKV infection to an increased GBS risk is the lack of a detailed characterization of the genomic variability of ZIKV associated with neurological diseases. It remains uncertain whether specific neurovirulent strains of ZIKV are responsible for the heightened susceptibility to neurological complications. However, the relationship between ZIKV infection during pregnancy and microcephaly, a birth defect characterized by an abnormally small head, has been clearly established [14]. This condition can cause serious neurological disorders in affected babies; and is the most devastating manifestation of the congenital ZIKV syndrome [15].

At present, ZIKV genome sequences from GBS cases have not been reported. In general, with GBS, it is often very difficult to prove that a given virus or bacteria caused GBS because neurological disease develops long after the infection has subsided. However, the most striking finding with ZIKV-associated GBS is that neurological symptoms were parainfectious after ZIKV infection, with prolonged viral shedding in urine [9,16]. Contrary to viral persistence in urine, ZIKV viral load in blood is cleared very early after the onset of ZIKV infection symptoms, both in those who develop GBS and those who do not develop this condition [17]. Therefore, urine samples are the best source for conducting viral-related studies, although previous to the Zika outbreak, it was not a mandatory biological sample for the clinical diagnosis of arboviruses and Zika only until the 2016 epidemic [18]. The Neurovirus Emerging in America Study (NEAS) working group established in Colombia for surveillance of emerging infections and neurological diseases maintains a repository of ZIKV-positive blood and urine samples from GBS patients and non-GBS Zika cases collected during the 2016 epidemic. Consequently, this study aimed to assess potential differences in the genomes of ZIKV-infecting individuals who develop GBS compared to those circulating ZIKV in the general population during the same time frame and in the same geographical locations.

## Results

### ZIKV genome sequencing in clinical samples

The genomes of ZIKV were obtained from urine and blood samples collected to 15 patients with GBS (ZIKV-GBS group) and 17 patients without any neurological symptoms (ZIKV-non-GBS group). Metadata and sample’s details are described in S1 Table and in Material and Methods section. Multiple comparative study samples were also available in three patients, including different biological sources (blood vs. urine), viral RNA origin (cell culture viral isolated *vs*. clinical sample, S1 Table). The average sequencing depth of coverage was nearly 3500X, showing differences between GBS (~2000X) and non-GBS samples (~5100X) (S1 Fig). The %GC content for both groups was similar, with a mean of ~51 for GBS and ~51 for non-GBS. The amplification scheme allowed for the recovery of 98% of the ZIKV genome for most samples, except for two cases where 90% and 91% were recovered (13740 and urine of 13738). The raw fastq files are available in the SRA repository at NCBI under accessions SRR26779677 to SRR26779712 and the assembled genome sequence (fasta) under accessions PP431214 to PP431251 (S1 Table).

A total of 216 Single Nucleotide Variants (SNV) were identified, with a Transition/Transversion ratio (Ts/Tv) of 8.14. Out of these, 183 (86%) were synonymous variants, 25 (12%) were non-synonymous variants, and 6 (3%) were located outside the polypeptide genes region. Most of the changes were observed in the NS5, NS3, and NS1 genes, with a lesser proportion in the pre/membrane (MP) and envelope (ENV) genes. Notably, these same genes exhibited the highest frequency of non-synonymous mutations (S2 Fig).

### Worldwide ZIKV phylogenetic tree

**Fig 1:**
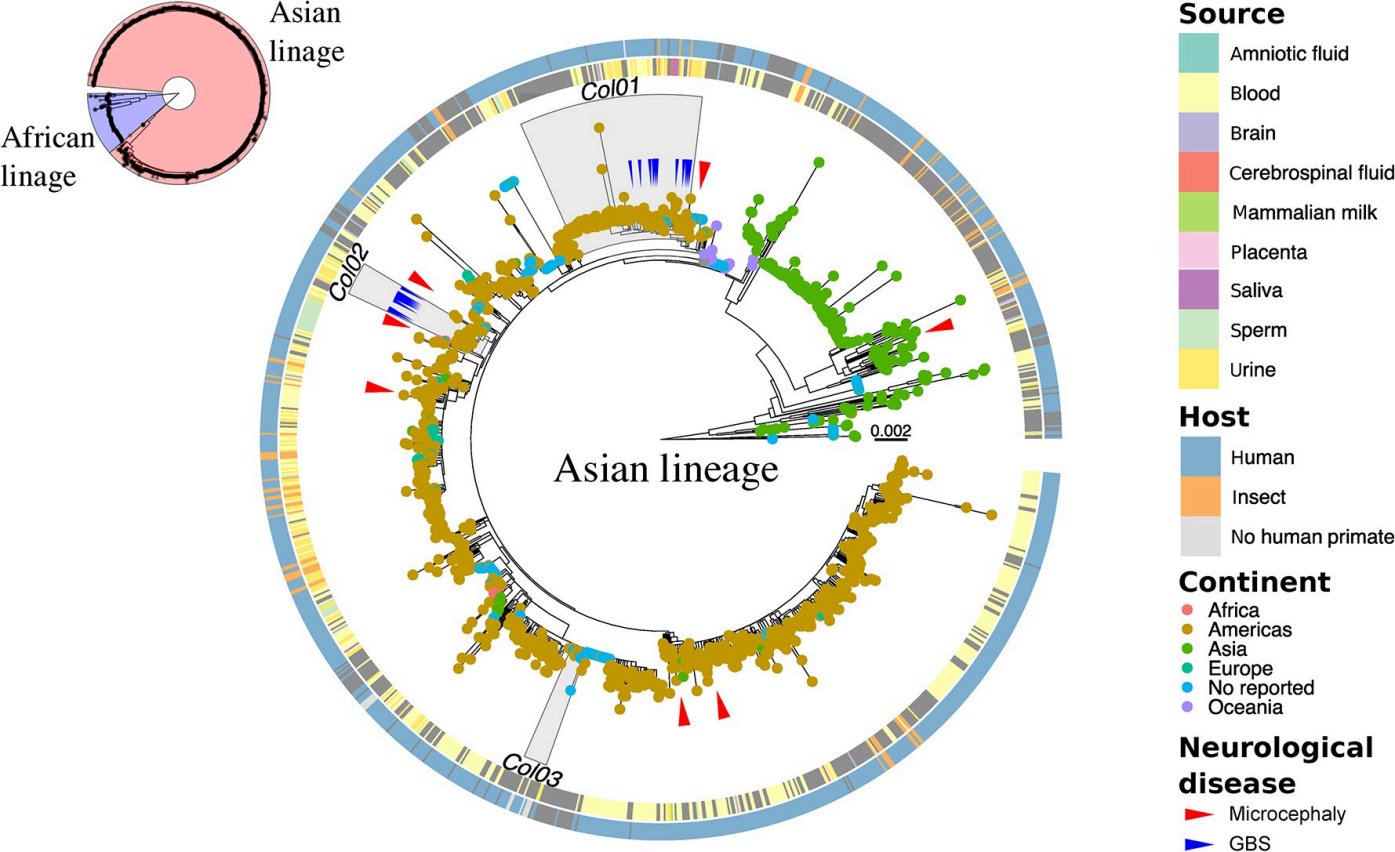
Maximum likelihood (ML) tree of the full database (1406 sequences) of genomes of Zika virus. In the upper left corner, all the sequences and the classification into the two main lineages (African and Asian) are represented. The central tree is a zoom of the sequences belonging to the Asian lineage. Color annotations are given in the circles around the terminal nodes (leaves). From inner to outer circle: 1) source of ZIKV isolation/sequencing; 2) Host of ZIKV isolation/sequencing. The color of the tips represents the continent from which each sequence was reported. The gray area indicates the three clades where the sequences for Colombia were grouped (Col01, Col02, and Col03). The red arrows indicate the position in the tree of microcephaly cases, and the blue arrows indicate the position of GBS cases.

A phylogenetic tree for ZIKV was constructed using the maximum likelihood method, which includes 1406 genomes collected worldwide from April 1947 to September 2021, including the ZIKV genomes reported in this study (Fig 1). This tree delineates two primary lineages: the African lineage, highlighted in blue and comprising 149 sequences, primarily representing older isolates spanning from 1947 to 2020, and the Asian lineage, highlighted in red with 1257 sequences, primarily derived from more recent viral isolates collected between 2006 and 2021. The Asian lineage is predominantly isolated from human hosts, with a lesser representation from mosquitoes, exhibiting greater variability and widespread distribution across Europe, Asia, Oceania, and the Americas. At the root of this clade, sequences from the French Polynesia outbreak in 2013–2014 hold a significant position, marking the initial instance where neurological complications, including GBS and microcephaly, were documented. ZIKV sequences from Colombia form a polyphyletic group, represented by three distinct clades (Col01, Col02, Col03). The Col01 clade exhibited the highest number of sequences (110) and the greatest average genetic distance (1.96x10^-3^ ± 1.17x10^-3^) among its members, followed by the Col02 clade with 20 sequences and an average genetic distance of 1.39x10^-3^ ± 5.96x10^-4^. Clade 03, on the other hand, was the least diverse, with only 14 sequences and an average genetic distance of 4.73x10^-4^ ± 9.44x10^-4^. These Colombian clades maintain a phylogenetic relationship with other ZIKV sequences from the Americas. Notably, most viral genomes from Colombia were obtained from urine or blood clinical specimens, while the clinical/host source of the Col03 clade viruses was not consistently reported.

### Comparison of ZIKV-GBS and ZIKV-Non-GBS strains

**Fig 2:**
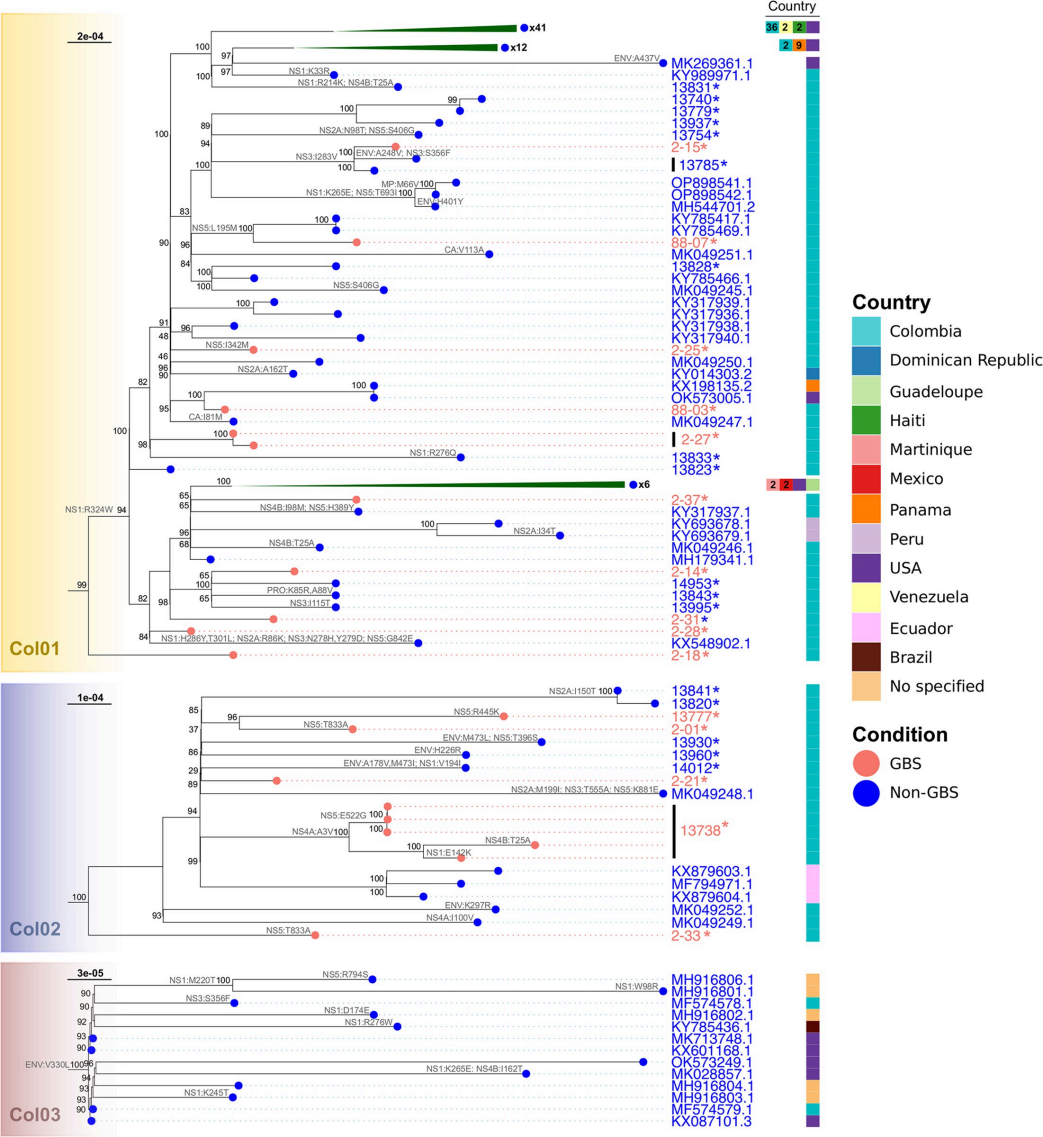
Representation of the three Colombian clades (Col01 to Col03) obtained of the ML tree. The color of the knots represents the clinical condition of the source from which the virus was obtained (Guillain-Barré syndrome: GBS or without neuroinflammatory complications: non-GBS). The annotations on the right side represent the country from which the sequence was reported, and the number within each box indicates the frequency in each country in cases >1. Branches with a vertical black line indicate that they belonged to the same patient but from a different source of isolation. Asterisk (*) indicates viral sequences from this study.

The ZIKV sequences obtained from GBS patients are dispersed throughout the Col01 and Col02 clades, as illustrated in Fig 2. Notably, no distinct clinical grouping patterns are discernible within these clades. In Col01, a monophyletic subclade encompasses viral strains 13740, 13779, 13754, 13937,13785, 2–15, OP898541.1, OP898542.1, and MH5444701.2, with only one corresponding to a GBS case (sample 2–15); this strain has minimal amino acid differences from other sequences in its group. It has one amino acid change in the NS3 gene (I283V), shared with strain 13785, a member of the non-GBS group. ZIKV-GBS 88–07 clusters within a distinct monophyletic subclade within Col01 along with the non-GBS strains KY785417.1 and KY785469.1, having the same amino acid sequence with just one difference in the NS5 gene (L195M) compared to nearby subclades. The ZIKV-GBS strains from case 2–27, originating from both urine and blood samples belong to the same monophyletic subclade with 13833 (non-GBS) which exhibited a distinct amino acid change NS1:R276Q. ZIKV-GBS 88–03 genome exhibits high similarity to ZIKV-non-GBS strains KX198135.2 and OK573005.1, with no observed amino acid changes. In contrast, the ZIKV-GBS 2–25 genome harbors a single amino acid change at position NS5:I345M. The ZIKV-GBS strains 2–37, 2–14, 2–28 and 2–31, which belong to a different subclade within the Col01 clade, also show no amino acid changes compared to their neighboring non-GBS strains, like 14953, MH1793341.1, 13995, and 13843. These neighbors have minimal amino acid changes, such as NS3:I115T, and PrM:K85R/PrM:A88V. Similarly, ZIKV-GBS strain 2–18, which lies on the most basal branch of the Col01 clade, is almost identical to other sequences in the same clade, except for the absence of the NS1:R324W mutation which is present in other members of the Col01 clade. The Col02 clade resembles the Col01 clade, as it lacks specific groupings of ZIKV genomes based on the GBS status of the host (Fig 2). Overall, most ZIKV strains exhibit only a few amino acid changes, with unique alterations in each sequence. Notably, among the ZIKV-GBS strains (2–01, 2–33, 13738, 13777) and in two non-GBS strains, 13930, MK049248.1, mutations occur in the NS5 gene, with the only shared mutation NS5:T833A in two ZIKV-GBS strains (2–33 and 2–01). Interestingly, ZIKV 2–21 is the only ZIKV-GBS strain within this clade without amino acid changes in the viral proteins. None of our ZIKV genomes cluster in Col03 clade that corresponded to 13 ZIKV-non GBS genomes all grouped by the unique change ENV:V330L.

ZIKV genomes obtained from multiple samples types from three individuals, two of them in the GBS group (2–27 and 13738) and one in the non-GBS group (13785) were compared to evaluate the results reproducibility. For ZIKV-GBS 2–27, two viral sequences were obtained (urine or blood) differing only in one synonymous change (c.4167C>T, S3 Fig). For ZIKV-GBS 13738 genomes from three clinical specimens, collected at different dates after GBS onset (Serum 02/01/2016, Plasma 02/04/2016, Urine 02/06/16), resulted in identical viral sequences to each other (S3 Fig) and shared the unique NS5:522G change (Fig 2). In contrast, the two *in vitro* isolated ZIKV strains (MiniBrain organoid and VERO cell line) from the same urine 13738 presented four nucleotide changes between them (S3 Fig) and the NS4B:T25A and NS1:E142K mutations depicted in Fig 2. These two *in vitro* generated ZIKV genomes differ from their viral counterpart in the original urine specimen by 6 and 4 nucleotides changes respectively (S3 Fig). Finally, for ZIKV-non-GBS 13785, both viral genomes from *in vitro* cultures (C6/36 HT cell line or MiniBrain organoid) accumulate unique nucleotide changes (3 and 1 changes respectively; S3 Fig), leading to ENV:A248V and NS3:S356F mutations in C6/36 HT cell line (Fig 2).

### Haplotype networks for fast evolutionary clustering of sequences

**Fig 3:**
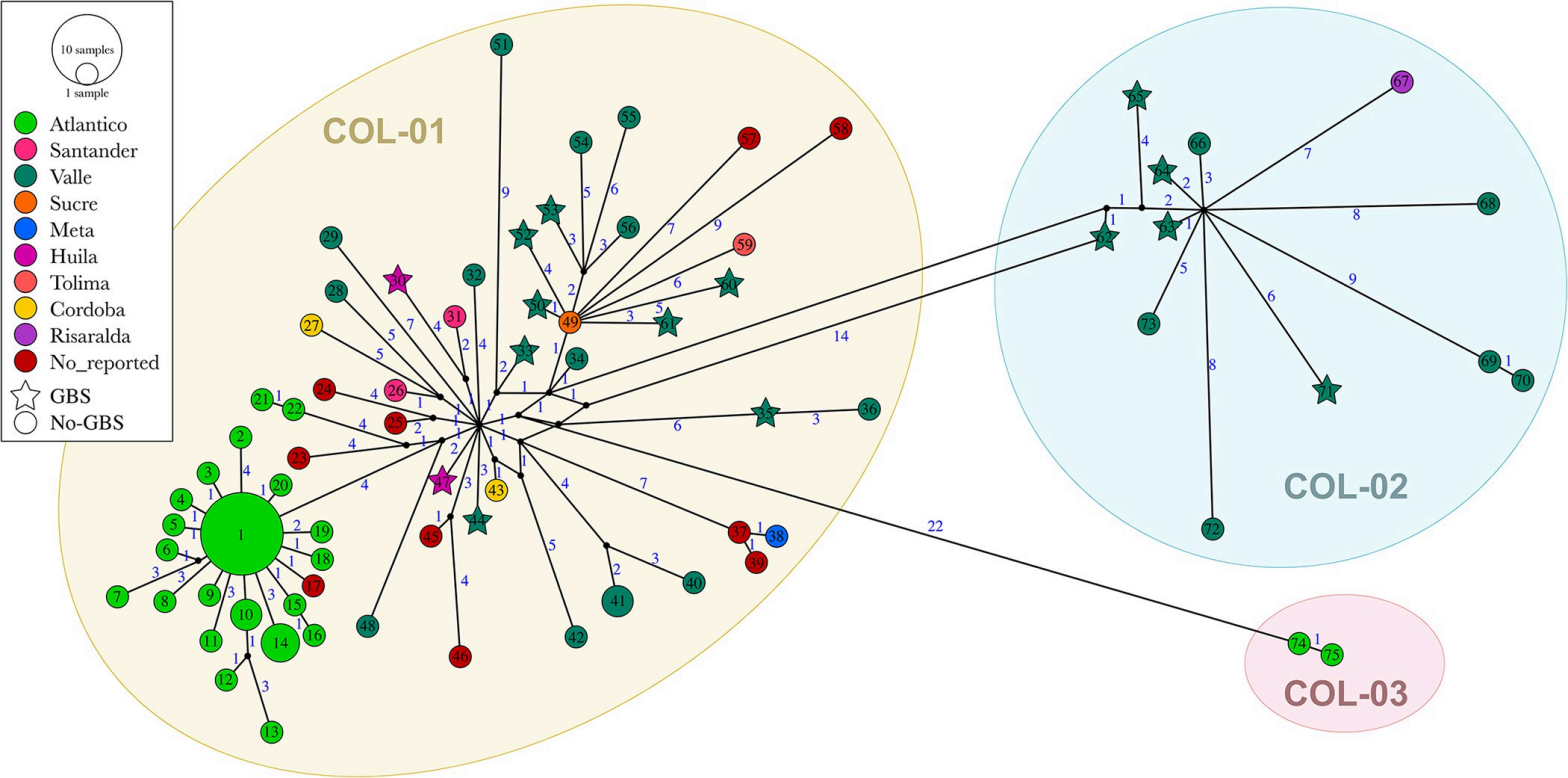
Haplotype network of Colombia ZIKV genomes. Each haplotype is represented by a circle (non-GBS) or star (GBS), the size is proportional to the frequency of each haplotype, and the color indicates the states of Colombia from which the sequence was reported. The length of the lines that join the haplotypes is proportional to the number of mutational steps between them, which are indicated with a blue number. The shaded areas indicate the haplotypes grouped in the phylogeny as Col01 to Col03.

We used a haplotype network to better understand the genetic diversity of the virus in Colombia. This approach helped us to identify different clustering patterns and viral population expansions across various regions of the country (Fig 3). A total of 77 haplotypes (S4 Table) were identified among 92 genomes, with the majority belonging to Col01 (63 haplotypes), followed by Col02 (12 haplotypes) and Col03 (2 haplotypes). Nucleotide diversity (Π) exhibited values of 1.38x10^-3^ for Col01, comparable to Col02, with a value of 1.43x10^-3^, but significantly lower for Col03, with a value of 9.95x10^-5^. Meanwhile, haplotype diversity (dH) was 0.97 for Col01 and 1.00 for Col02 and Col03. Interestingly, ZIKV genomes associated with GBS did not exhibit distinct clustering or stem from viral population expansions; instead, they were dispersed within the network, characterized by star-shaped haplotypes (Fig 3). Col01 haplotypes demonstrated a broader geographical distribution, spanning the Colombian states of Atlántico, Córdoba, Valle del Cauca, Sucre, Huila, Meta, Santander, and Tolima. This group revealed a network complexity value (HBd) of 0.60, with observable subgroup expansions, particularly in Atlántico and Valle del Cauca. Col02 predominantly comprised viruses from Valle del Cauca and one from Risaralda, displaying a star-shaped expansion pattern with an HBd value of 0.40. In contrast, Col03 only had two members from Atlántico, with an HBd value of 0.00.

### Analysis of molecular variance of the ZIKV

The analysis of molecular variance (AMOVA) (Table 1) indicated that there were significant differences (p < 0.001) when the ZIKV genomes were grouped by states within Colombia. This resulted in an Φst value of 0.24. However, a greater portion of the variability was explained when the genomes were grouped by phylogenetic clades (Col01, Col02, Col03). This grouping explained 60.09% of the total variability of Colombian genomes, with an Φst value of 0.60 (a statistically significant difference with p < 0.001). This grouping trend persisted even when only the genomes reported in this study were considered, resulting in an Φst value of 0.57 and a p-value < 0.001.

**Table 1:** Molecular analysis of variance (AMOVA) of ZIKV for Colombia.

Source of variation	d.f.	Sum of squares	% variation	Fixation indices	p-value
**Per states of Colombia**					
Among states	9	4710.72	23.75	Φst: 0.24	< 0.001
Within states	82	10256.70	76.25		
Total	91	14967.42	100.00		
**Per clades (Col01, Col02, Col03)**					
Among clades	2	9163.35	60.09	Φst: 0.60	< 0.001
Within clades	89	5804.07	39.91		
Total	91	14967.42	100.00		
**Per conditions (GBS Vs Non-GBS)**					
Among conditions	1	817.66	4.37	Φst: 0.04	0.003
Within conditions	90	14149.76	95.63		
Total	91	14967.42	100.00		
**Per condition (GBS Vs Non-GBS, only genomes of this study)**					
Among conditions	1	-11.58	-3.34	Φst: -0.03	1.000
Within conditions	30	9832.55	103.34		
Total	31	9820.97	100.00		
**Per clades (Col01, Col02, Col03, only genomes of this study)**					
Among clades	2	6445.97	57.09	Φst: 0.57	< 0.001
Within clades	31	4230.94	42.91		
Total	33	10676.91	100.00		

In contrast, when the ZIKV genomes from Colombia were assessed according to the neurological condition (GBS vs. Non-GBS), only 4.4% of the explained variation was observed (Φst: 0.04), although the p-value remained significant (0.003). To avoid potential bias, a similar test was conducted exclusively on genomes collected in this study. This revealed an absence of structuring in ZIKV genomes from patients with GBS and those circulating in the same city (Φst: 0.04; p-value: 1.00).

## Discussion

This study aimed to investigate whether specific ZIKV genotypes were associated with the outbreak of ZIKV-associated GBS in Colombia. We analyzed ZIKV-positive urine samples collected during the 2016 ZIKV outbreak in Colombia from both GBS cases and controls without neurological symptoms. We conducted full genome sequencing using Oxford-Nanopore technology on ZIKV samples from 15 GBS cases and 17 non-GBS controls.

Our findings revealed that the ZIKV genotypes in Colombia could be grouped into three distinct clades. Two of these clades contained most of the samples. Despite this diversity, a comparative analysis of ZIKV genomes from GBS cases and non-GBS controls, including 1,368 previously reported genomes, found no significant genetic differences between the groups. The distribution of ZIKV genotypes among the Colombian clades was consistent across both GBS and non-GBS cases. Furthermore, the amino acid sequences in the ZIKV genomes from GBS and non-GBS cases were highly similar, with no significant variations that could be linked to the development of GBS. These results suggest that specific ZIKV genotypes or amino acid variations are unlikely to be the primary factors in GBS development, indicating that other mechanisms might be responsible for the neurological symptoms seen in some ZIKV-infected patients.

The physiopathology of GBS includes an abnormal immune response suspected to be of autoimmune origin, targeting peripheral nerves and spinal roots generally 1 to 2 weeks after a preceding infection. GBS has been classified as axonal and demyelinating forms, the humoral immunity in both clinical forms is against nodal (N)/paranodal (PN) and internodal (IN) regions of the axonal-myelinic region epitopes in association with the complement system activation and formation of the membrane attack complex (MAC) which lead to acute damage [3]. Molecular mimicry mechanisms have been suggested for the microorganisms associated with the development of GBS. The glycans present in the lipo-oligosaccharides of some *Campylobacter jejuni* serotypes contain ganglioside mimicking structures including GM1, GM2, GD1a, GT1a, and GD3 and an immune response against *Campylobacter* may later cross react with peripheral nerve gangliosides [19,20]. It is not clear at what extend ZIKV-GBS share similar anti-ganglioside and other antibody signatures as in the classical forms of GBS. There is biological plausibility for molecular mimicry between viral and host epitopes, which has been proposed as a potential mechanism driving various non-GBS neuroinflammatory diseases in humans. For example, neuromyelitis optica spectrum disorder (NMSOD) may involve cross-reactive epitopes between human aquaporin-4 and the Tax protein of the Human T-lymphotropic virus type 1 (HTLV-1). Similarly, in multiple sclerosis (MS), cross-reactive antibodies or T cells have been identified between epitopes of the Epstein-Barr virus Nuclear Antigen -1 (EBNA-1) and antigens of the central nervous system (CNS) [21,22].

### Viral genomes and quality control

This research represents the first reporting of ZIKV genomes in patients diagnosed with GBS (%GC content ~50.1 and average depth of coverage ~3400X). To date, only seven ZIKV genomes linked to microcephaly have been documented [23–28] (Fig 1), with none associated with GBS or encephalitis. Our study compared the reproducibility of ZIKV sequencing across multiple samples obtained to the same case and the concordance between sequencing from clinical specimens and viral *in vitro* isolates (VERO and C6/36 HT cell lines, MiniBrain organoids) (S3 Fig). High reproducibility was observed between multiple genome sequences obtained from the same case; however, mutations occur in the replicating virus in cell cultures or the organoids compared with viral genomes from the original biological samples (serum, plasma, or urine). For ZIKV-GBS 2–27, only one difference was observed between the genomes obtained from blood and urine samples (S3A Fig). In the case of ZIKV-GBS 13738, samples of clinical origin were very similar to each other, but the *in vitro* cultured ZIKV samples (VERO) or MiniBrains presented distinct mutations, some of which were exclusive (S3B Fig), highlighting the bias that viral replication *in vitro* can introduce in the accumulation of mutations. This same behavior was observed with ZIKV-non-GBS 13785 (S3C Fig). Therefore, targeted PCR enrichment of the viral RNA in the clinical sample proved to be an appropriate strategy for ZIKV genome completeness as it avoids additional *in vitro* culture steps, reduces the presence of artifacts in sequence acquisition, increases speed of processing, and lowers costs.

### Phylogenetic tree of ZIKV GBS and non-GBS

Our study found no significant differences in the ZIKV genomes between ZIKV-infected patients with GBS and without GBS. A comparative analysis of the genomes from both groups, alongside 1368 previously reported ZIKV genomes, revealed a similar distribution of genotypes across the three identified clades in Colombia. Notably, we did not detect any variations in the amino acid sequences encoded by the viral genome in cases of ZIKV-associated GBS compared to those without GBS or the broader pool of ZIKV genomes.

Consistent with earlier studies [29], our research confirms the presence of two distinct lineages: the African and Asian lineages. The Asian lineage, which originated in Oceania, reached the Americas through a single introduction in Brazil, setting off an epidemic that spread across the continent [29]. After this initial entry, ZIKV spread to several countries in northern South America, encompassing Colombia, Venezuela, Suriname, and French Guiana. In Colombia, the introduction of ZIKV occurred through multiple separate entries from different clades, leading to localized outbreaks (Fig 1). This process gave rise to three different clades, with one showing the highest diversity and prevalence in the country. This pattern aligns with the findings in the study by Black *et al*. [30], which also identified two primary clades as dominant in Colombia. Notably, most of the ZIKV diversity in Colombia can be traced to a single introduction. The Col01 and Col02 clades, which primarily consist of samples from Colombia, underscore the crucial role in the virus’s spread within the country. In contrast, the Col03 clade is more frequently reported in the USA, indicating that its introduction in Colombia had a less significant impact on the local transmission of ZIKV. This observation highlights those different clades can have varying levels of influence on the spread of the virus in specific geographical regions (S4 Fig).

Given that no microcephaly or GBS cases were reported among earlier ZIKV infections before the French Polynesia epidemic in 2014 further spreading to America and the Caribbean region, one hypothesis is that specific pathogenic molecular variants may had contributed to ZIKV neurovirulence [31–33]. Therefore, we aimed to investigate whether certain ZIKV genomic variations could help explain why some ZIKV-infected individuals develop GBS while others do not. When examining the phylogenetic distribution of ZIKV from GBS patients, we found no evidence of a distinct clustering pattern among these cases, nor did they originate from a unique phylogenetic clade separate from most circulating viruses in Colombia. The spread of ZIKV-GBS sequences across various prevalent clades in Colombia indicates that these strains are neither linked to rare lineages nor geographically confined within the country. This observation challenges the notion that ZIKV-associated GBS has a localized or lineage-specific basis, suggesting instead that the development of neurological complications like GBS involves a more complex set of factors, potentially related to the host or immune response.

An examination of the ZIKV clades in Colombia reveals that ZIKV strains from GBS patients do not consistently share recent substitutions, either within their group or across other GBS cases. Similarly, many of the trunk molecular variants are shared with isolates causing infections that do not lead to GBS or other neurological complications. This pattern was particularly noticeable in the clade Col01, where all ZIKV isolates from GBS cases showed no unique substitutions in the tree’s branches, suggesting no common changes among these cases. In contrast, in clade Col02, a few distinct amino acid changes were noted for each GBS sample. Notably, most changes in this clade were concentrated in the NS5 gene, consistent with its extensive size, comprising 903 amino acids in the ZIKV genome [34]. This gene plays a pivotal role, encoding a viral protein with diverse functions, including viral RNA synthesis and modulation of the host’s immune response [34]. Specifically, the NS5:R445K alteration in ZIKV-GBS 13777 stands out as unique. However, transitioning from a basic amino acid (R) to another basic amino acid (K) may impart a relatively modest impact on protein function. Additionally, the NS5:T833A change, observed as a convergence in distant branches of ZIKV-GBS strains 2–01 and 2–33, is evolutionarily recurrent, extending to the general trunk of Col01, encompassing numerous non-GBS samples from the same city, and sequence MW165882.1 situated in another distant clade from the USA. ZIKV isolates from the GBS-case 13738 consistently presented the NS4A:A3V mutation across various sample sources, clinical and *in vitro* cultures, alongside the NS5:E522G change unique to the viral isolates from clinically-derived samples. The shift from an acidic amino acid (glutamate) to a neutral residue (glycine) may influence electrostatic interactions and protein structure of NS5, the viral RNA polymerase. Interestingly, this ZIKV strain was isolated from a case of GBS with a fatal outcome and prolonged viral excretion. Furthermore, NS1:E142K mutation was exclusive to this ZIKV 13738 when growth in VERO cells, suggesting this amino acid change might occur during the viral replication *in vitro*. In contrast, an NS4B:T25A change induced when the same virus replicated in MiniBrains demonstrated high convergence, appearing in viruses from Tolima and Valle del Cauca within Col01, as well as in several samples from Brazil and Nicaragua belonging to other distant clades, regardless the neurological status of the source. Remarkably, ZIKV-GBS 2–21 exhibited no changes as the other members of the clade. These observations reject the assertion that these amino acid substitutions are critical for developing neurological complications such as GBS, although their importance remains uncertain.

## Comparison with previously reported ZIKV mutations

Various studies have examined the impact of non-synonymous mutations in the ZIKV infectivity, replication rates, and neuropathogenicity in mice, mosquitoes, or cell cultures. Yuan *et al*. [32] found that the PrM:S17N mutation increases ZIKV infectivity in human and mouse neural progenitor cells and promotes apoptosis. The S allele, representing the original version, is common in Africa, India, and Southeast Asia, where no neurological complications of Zika have been reported, while the N allele, reflecting the mutation associated with greater infectivity *in vitro*, is found in China, Russia, and the Americas including Colombia regardless the neurological condition, except the genome KY317939.1 with a unique PrM:S17K mutation. Therefore, PrM:S17N mutation might not be a direct cause of GBS. A mutation in NS1 protein (NS1:A188V), seems to also contribute to enhancing ZIKV infectivity in laboratory *A*. *aegypti* mosquitoes [35] and neonate mice brains by interfering with IFN-β pathway [36]. This mutation has been observed naturally in ZIKV isolates from cases in Southeast Asia and America but not in Colombia. The ENV:V473M mutation increases ZIKV replication, resulting in enhanced neurovirulence, greater mother-to-fetus transmission, and elevated viremia in non-human primate and mouse models [37]. This mutation is predominant in the Asian lineage but not in the African lineage with ZIKV genomes from Southeast Asia exhibiting this mutation, whereas it is absent in the Americas and Colombia.

Two additional mutations, CA:T106A and NS5:M872V, have a lesser effect on ZIKV replication [32,38] and are present in ZIKV strains worldwide except in Southeast Asia. The NS2B:I39T mutation, known to also increase the ZIKV replication in human neural precursor cells and laboratory-reared mosquitoes [39], is found only in viruses from Indonesia and Japan but absent in our Colombian ZIKV sequences. Fontes-Garfias *et al*. [40] and Carbaugh *et al*. [41], reported that the mutagenesis-induced ENV:N154Q change, crucial for the glycosylation of the viral envelope, had a significant effect on ZIKV neurovirulence in A129 mice. However, this mutation has not been observed in any naturally transmitted ZIKV genome in humans. Similarly, the ENV:67N mutation, associated with increased viral production and severe cytopathic effects in human neural astrocyte cells, reported by Z. Liu *et al*. [42], is not a natural viral variant.

Collette *et al*. [43] identified a set of mutations—PrM:V1A, NS1:G100A, NS2B:M32 (with derived alleles I/V/T/L), NS3:M572L, NS3:Y584 (with derived alleles H/C/R)—that were associated with decreased replication in mammalian cells, increased embryonic death, and developmental defects. NS1:G100A and NS3:M572L primarily occurred in samples from Nicaragua and Mexico. The NS3:Y584H mutation was common across the Americas, including all Colombian genomes, suggesting a broader geographical distribution. The NS2B:M32 mutations had various derived alleles (I/V/T/L), with the I allele found in two Colombian samples (MF574585.1 and MF574587.1), none of which were linked to GBS. The PrM:V1A mutation is only observed in some Southeast Asian samples but not in the Americas or Colombia.

Zhang *et al*. [38] analyzed the ZIKV genomes from Cambodia in 2010 and 2019, identifying 12 amino acid changes, some of which have been associated with neurovirulence. The changes CA:T106A, PrM:V1A, PrM:S8N, PrM:M29L, NS1:A188V, NS2A:P128L, NS5:V358A, NS5:M872V, and NS5:V883M were prevalent in Asian countries like Thailand, India, Cambodia, South Korea, Micronesia, or Indonesia but are not found in Colombian ZIKV genomes, and only in few cases in the Americas. In contrast, ENV:V473M and NS2B:A105T were present across the entire Asian lineage and were present in all Colombian sequences. Position 58 in NS2A protein showed multiple alleles (NS2A:A58 and derived alleles T/V/I) with the T allele linked to a microcephaly case in Thailand but also present in viral genomes from cases not related to microcephaly in Thailand, Japan, and Cambodia and absent in the Colombian clades. In comparison, the V allele is typical of the African lineage.

Overall, despite *in vivo* and *in vitro* evidence suggesting that certain mutations in the ZIKV genome can affect virulence or neuropathogenicity, only a few of these mutations were identified in our dataset, regardless of the neurological condition. No specific viral mutations were found to distinguish GBS associated ZIKV genomes from non GBS ZIKV genomes, nor were any associated with viral load or prolonged ZIKV shedding. However, prolonged viral positivity is a hallmark of ZIKV-associated GBS [9]. The viral loads in ZIKV-GBS urine samples (mean CT = 28.8 ± 4.6) were persistently high after Zika symptoms (mean days = 22.8 ± 14.9), comparable to the viral levels in ZIKV-non-GBS samples (mean Ct = 25.6 ± 3.4) collected earlier during the first week of Zika disease onset (mean days = 4.7 ± 2.1), as depicted in S1 Table. There was no evidence of prolonged ZIKV positivity in the ZIKV-non-GBS cases (metadata not included).

### Haplotype network and AMOVA of Colombian ZIKV

The haplotype network of Colombian ZIKV genomes revealed distinct grouping patterns and indicated different dynamics of virus spread across the country (Fig 3). An analysis of molecular variance (Table 1) demonstrated a high level of genetic structure (Φst: 0.60) when sequences were grouped based on the clades defined in the phylogenetic tree. However, when analyzing the ZIKV genomic variation by geographical location (Colombian States), the structure variance was lower (Φst: 0.24, p<0.001) yet still statistically significant. AMOVA by GBS condition showed low significant structure (Φst: 0.04, p<0.05), due to a bias introduced by ZIKV-GBS genomes all originating from the same state (Valle del Cauca) whereas the non-GBS genomes were from multiple geographical locations. However, when the comparison by GBS condition was limited to ZIKV genomes within the same stated the structure was negligible (Φst: -0.03, p = 1.0). These findings suggest that the genetic variation observed in ZIKV genomes in Colombia is not significantly associated with GBS condition, but is primarily driven by the overall viral spread within the country.

In summary, our study investigated the association between ZIKV genomic variation and Guillain-Barré syndrome in Colombian patients. ZIKV-GBS genomes were distributed across two of the three major ZIKV clades circulating in Colombia. Importantly, we found no significant differences in clade composition between GBS and non-GBS cases. Additionally, no specific amino acid changes in the viral proteins were associated with GBS cases compared to controls. These findings suggest that genetic variations within ZIKV itself are unlikely to explain the development of GBS following ZIKV infection. Instead, our study highlights that perhaps other potential host factors play a more critical role in GBS pathogenesis. This underscores the need for further research into host-virus interactions and immune mechanisms to elucidate the cause of ZIKV-associated neurological complications.

## Materials and methods

### Biological samples and retrospective cohort

The study comprised retrospective clinical samples (urine n = 15 and serum (n = 3) from 15 patients diagnosed with GBS according to Brighton’s criteria during the 2016 ZIKV outbreak [44,45], and confirmed positive for ZIKV through RT-PCR analysis [9], designated as the ZIKV-GBS group. Additionally, 14 urine specimens, two serum samples, and one low pass *in-vitro* viral isolate (C6/36 HT cell line) from blood were processed from 17 subjects who tested positive for ZIKV during the same timeframe but did not display any neurological disorders, referred to as the ZIKV-non-GBS group. In addition, two samples (13738 ZIKV-GBS and 13785 ZIKV-non-GBS) first isolated in VERO and C6/36 HT cell lines respectively were further cultured in MiniBrain organoids, and were used as controls (S1 Table). These samples were obtained during the 2016 ZIKV outbreak as part of the Neuroinfections Emerging in the Americas Study (NEAS), which enrolled patients across various institutions in several states in Colombia, including the cities of Cali and Neiva (S1 Table), where samples analyzed in this study were selected from. The NEAS project incorporated comprehensive clinical and neurological assessments conducted by internal medicine and neurology specialists with full characterization of the cases previously reported [9]. Briefly, the neurological symptoms at presentation included limb weakness, and paresthesias or facial palsy and most cases reported an ascending pattern of weakness (S1 Table). All samples were accessed from June 8, 2022, to November 19, 2022, from the NEAS Biorepository at Universidad del Valle. Johns Hopkins University safeguards participant information on the RedCap platform, ensuring the anonymization of samples and data. Study researchers only had access to diagnosis type (GBS vs. non-GBS), age, and sex, with no access to identifiable information. The study was approved by the Ethics Committee of Universidad del Valle, Colombia, with code 034–016.

### ZIKV RNA extraction

Viral RNA was extracted from clinical samples (29 urine and 5 serum samples; S1 Table) and four viral *in vitro* isolates (2 in MiniBrain organoids and 2 in cell lines VERO or C6/36; S1 Table) using either the QIAamp Viral RNA kit (Qiagen, Hilden, Germany) or the MagMAX™ Viral/Pathogen Nucleic Acid Kit for KingFisher Flex (Thermo Scientific, Waltham, MA, USA), following the respective manufacturer’s instructions. After extraction, eluted RNA was stored at -80°C until further processing. The presence of the ZIKV virus was determined through quantitative RT-qPCR, targeting two genomic regions (E and NS2B) as per established protocols [46,47]. The human RPP30 gene served as the RNA control [48] (S2 Table) in clinical specimens. A sample was considered positive for ZIKV only if both genomic target regions in RT-qPCR returned positive results. For sequencing purposes, ZIKV-positive RNA samples with CT values below 35 (S1 Table), displaying a typical sigmoid amplification curve, were selectively considered.

Clinical samples of different fluid types duplicates (urine and blood) from cases 2–27 and 13738, diagnosed with GBS, were included for comparison purposes. Additionally, for sample 13738, RNA samples from *in vitro*-isolated viruses in VERO cells and MiniBrain organoids (obtained according to the *in vitro* procedures described below) were available for sequencing. The goal was to evaluate the reproducibility of the sequencing results between different biological samples within the same individual and to assess the concordance of the genomic sequences performed directly from clinical samples versus viral cultures.

Previous studies suggested that ZIKV detection in urine is more sensitive and has a longer detection window compared to other biological fluids [16,49,50]. Therefore, our study primarily utilized this type of sample to enhance the likelihood of obtaining sufficient viral copy numbers for adequate sequencing.

### Cell lines and viral culture

VERO cells (E6, ATCC®) using Dulbecco’s modified eagle medium (DMEM, Gibco, Grand Island, NY, USA) supplemented with 2% bovine serum albumin (BSA, Gibco), were inoculated with a ZIKV PCR positive urine sample (13738) sourced from a GBS patient during the 2016 epidemic in Colombia (S1 Table) to isolate the ZIKV; After 5 days in culture at 37°C and 5% CO_2_ concentration ZIKV was isolated, and the positive culture supernatant re-inoculated in a new VERO cell monolayer to expand the infectivity to 80% cytopathic effect (CPE). This second passage of ZIKV in VERO cells was used for RNA sequencing by Oxford nanopore and for infecting an organoid MiniBrain system [51,52]. C6/36 HT mosquito cells were also infected with the serum of a non-GBS ZIKV positive case (13785) and incubated at 34°C until the typical flavivirus CPE appearance under inverted microscopy. Culture supernatant was harvested at 5 days postinfection (dpi) and stored at −80°C until use for RNA extraction and sequencing by Oxford nanopore or for infecting the 3D MiniBrain system and further sequenced by Illumina.

An iPSC-derived 3D MiniBrain model was developed that consists of human neuronal and glial cell populations including GABAergic, glutaminergic, and dopaminergic neurons, astrocytes, and oligodendrocytes [51]. At 6 weeks of development, the MiniBrain organoids were infected with the above-described low passage cell culture isolated ZIKV strains. The MiniBrains organoids were collected one week post-infection and the ZIKV infection was confirmed by RT-PCR, immunohistochemistry, and electron microscopy. Culture supernatants of the ZIKV-positive MiniBrains, designed as S8 (ZIKV-GBS) and S7 (ZIKV-Non-GBS) viral isolates were used for RNA extraction and further processed for RNAseq by Illumina.

### Illumina MiSeq protocol

Viral RNA was extracted from S8 and S7 viral isolates using the miRNeasy Micro Kit (Qiagen, Valencia, CA, Catalog #217084) following the manufacturer´s instructions, and then sequenced using the Illumina HiSeq 2500 System (Illumina Inc., San Diego, CA, USA), paired-end (150bp x 2) at the Genetic Resources Core Facility (GRCF), Johns Hopkins, using standard Illumina protocols. The output for the samples was > 4 million reads.

### cDNA synthesis and tiling-based polymerase chain reaction for ONT sequencing

The synthesized cDNA was performed from total RNA using the LunaScript® RT SuperMix kit (New England Biolabs, Ipswich, MA, USA). We used 8 μl of eluted RNA and 2 μl reverse transcriptase according to the manufacturer’s specifications. RT was performed at 25°C for 2 min, 55°C for 10 min, and 95°C for 1 min. cDNA was enriched through a tiling amplification strategy using Q5 hot start high-fidelity (New England Biolabs, Ipswich, MA, USA).

The amplification process adhered to the protocol and primer scheme outlined by Quick *et al*. [53], conducted in two separate PCR reactions: PCR Pool 1 (36 primers) and PCR Pool 2 (34 primers) (Integrated DNA Technologies, Leuven, Belgium). However, to enhance the depth coverage of the whole ZIKV genome, specifically to correct amplification and sequencing problems presented between positions 4925 and 7019, additional primers were custom-designed using PrimalScheme software [53] and used as a supplement to the PCR reaction. Seven new primers for Pool 1 and six for Pool 2 were developed based on eight local Colombian whole ZIKV genomes obtained in the ONT standardization and two preliminary Illumina RNAseq ZIKV genomes (S3 Table), which achieved a recovery rate of 98.0% of the viral genome (S1 Fig).

PCR amplification was carried out using the following program: initial denaturation for 30s at 98°C, followed by 35 cycles of denaturation for 15s at 98°C, and 5 minutes of annealing and extension at 63°C. Amplicons of approximately ~450 bp were generated. Subsequently, 10 μL of Pool 1 and 10 μL of Pool 2 PCR products for each sample were combined (Pool 1/Pool 2) and subjected to a 1:2 dilution. The quantification of these diluted products was performed using a Qubit fluorometer with the Qubit™ dsDNA HS Assay (ThermoFisher Scientific, Waltham, USA).

### ONT library preparation and sequencing of ZIKV genomes

Sequencing libraries were prepared using the Native Barcoding Kit 96 (SQK-NBD112.96, Oxford Nanopore Technologies). The PCR amplicons were repaired and A-tailed using the NEBNext® Ultra™ II End Repair/dA-Tailing Module kit (New England Biolabs, Ipswich, MA, USA). Native barcodes and adaptors were ligated to amplicons using the NEBNext Ultra II Ligation Module (New England BioLabs). The barcoded samples were pooled and purified using 0.7X AMPure XP beads (Beckman Coulter; Brea, CA), and later quantified using Qubit™ dsDNA HS assay (ThermoFisher Scientific, Waltham, USA). Adapters were ligated to the pooled library using adapter mix II (AMII H; Oxford Nanopore Technologies), NEBNext® quick ligation reaction mix (New England Biolabs, Ipswich, MA, USA), and Quick T4 DNA ligase (New England Biolabs, Ipswich, MA, USA) by incubation at room temperature for 20 min. The adapter-ligated libraries were cleaned up using 1X AMPure XP (Beckman Coulter; Brea, CA) were performed. The final libraries (78–110 ng DNA) were sequenced on an R10.4 flow cell using a MinION Mk1B or Mk1C device with MinKNOW Ver.22.12.5 software, according to the ONT protocol for 48 h or 72 h run.

### Bioinformatic processing

#### Basecalling, demultiplexing, and barcode trimming

The base-calling, demultiplexing, and barcode trimming of raw data (.fast5) were processed using the Guppy v6.5.7 in the GPU mode with parameters guppy_basecaller—flowcell FLO-MIN112—kit SQK-NBD112.96—min_qscore 8. Genome assembly was conducted with the ARTIC Network v.1.2.1 pipeline (https://artic.network/ncov-2019/ncov2019-bioinformatics-sop.html), using the recommended configuration. The ARTIC pipeline was also modified to utilize the ZIKV primer scheme (S3 Table), and KJ776791.2 reference genome as outlined in https://github.com/zibraproject/zika-pipeline. Quality control and read filtering were performed using the Artic guppyplex filter, focusing on reads within the 400 to 3000 bp range; this length filtering strategy aims to exclude chimeric reads and short fragments. Variant analysis and consensus sequence generation were performed using Medaka v1.11 included in Artic (https://github.com/nanoporetech/medaka); sites with a depth lower than 50× were masked by N bases, and the reference was substituted by homozygous variations with a Phred quality≥20. Sequencing depth and coverage were evaluated using QualiMap v2.2.2. [54] and QUAST [55], respectively.

## Review and quality control of sequences

To mitigate potential cross-contamination in high-throughput sequencing experiments, an "external contamination control" was implemented, as suggested previously [56]. This allowed for establishing the sequencing depth threshold for deeming a sequence reliable. External contamination controls were amplified for metabarcoding of the 16S rRNA and 18S rRNA genes (S1 File). The threshold was set as the average mapping depth value for the ZIKV genome obtained in external contamination control samples. ZIKV genomes below this threshold were excluded from subsequent analyses.

Consensus genomes underwent a thorough visual inspection for ambiguous bases, indels, and overall genome integrity. Manual adjustments were made as needed. Each single nucleotide variant (SNV) or indel was verified using IGV v2.16.2 [57] and the corresponding mapped files (.bam). Identified errors were corrected, and any ambiguities that couldn’t be confirmed or rectified were designated as "N."

## Identification of molecular variants and phylogenetic analysis

ZIKV genomes obtained in this study were compared with 1368 full-genome sequences available in the NCBI public database (accessible at: https://www.ncbi.nlm.nih.gov/labs/virus/vssi/#/virus?SeqType_s=Nucleotide&VirusLineage_ss=Zika%20virus,%20taxid:64320, consulted on November 8th, 2023), with a size of at least 90% of the reference genome (≥9700bp), and originating from diverse geographic regions. Phylogenetic analysis was conducted using the Nextstrain bioinformatics pipeline [58]. The pipeline encompasses multiple sequence alignments performed with MAFFT [59], maximum likelihood phylogeny inference using IQ-TREE [60], and molecular clock calibration with TreeTime [61]. The results, in JSON format, were interactively explored on the auspice.us web platform. The phylogenetic tree and annotations were generated using the ggtree [62] and ggtreeExtra [63] packages.

## Haplotype networks and sequence comparison

A sequence comparison for Colombia was conducted by constructing haplotype networks with the Median-joining networks algorithm [64] in the popArt v.1.7 software [65]. Nucleotide diversity indices (π), haplotype diversity (dH), and Haplotype Network Branch diversity (HBd) were calculated using the scripts described by Garcia *et al*. [66]. A one-level Analysis of Molecular Variance (AMOVA) in the PopArt v.1.7 [65] was performed to evaluate the effect of clades, state grouping, or neurological condition (GBS Vs non-GBS) on ZIKV genetic variation in Colombia. Likewise, a two-level AMOVA was conducted to assess ZIKV genetic variation concerning neurological conditions within the observed clades for Colombia.

## Supporting information

S1 Table Metadata of samples included in the study, sequencing method, and NCBI accession. (PDF)

S2 Table Primers and probes used for ZIKV detection by RT-qPCR. (PDF)

S3 Table ZIKV primers scheme. (PDF)

S4 Table Haplotypes list. (PDF)

S1 Fig Basic information on genome assemblies. Descriptive of the assembled genomes of depth coverage mean, %GC, mapping quality, coverage, and cycle threshold (CT) of sequenced ZIKV genomes for the GBS and non-GBS groups.
(TIF)

S2 Fig Comparison of synonymous and non-synonymous mutations of ZIKV gene between patients with GBS and non-GBS. The size of the circles is proportional to the frequency of mutations with respect to the reference genome KJ776791.2. The cyan color indicates non-missense mutations, and the red color indicates missense mutations.
(TIF)

S3 Fig Venn diagrams of different samples from the same case. **A.** Comparison of ZIKV sequences of urine and blood samples from ZIKV-GBS 2–27 obtained by Nanopore. **B.** Comparison of ZIKV sequences obtained from blood (plasma and serum), urine, cell culture in VERO (Vp2) sequenced by Nanopore, and MiniBrains organoids sequenced by Illumina from ZIKV-GBS 13738. **C.** Comparison of ZIKV sequences from ZIKV-non-GBS 13785 that were cultured in C6/36 cells and sequenced by Nanopore or cultured in MiniBrains and sequenced by Illumina. All mutations are compared to the reference genome KJ776791.2.
(TIF)

S4 Fig Mapping ZIKV spread in Colombia. Geographic spread of the Zika virus (ZIKV) in Colombia during the 2016 outbreak. Each circle represents a state within Colombia where ZIKV sequences have been reported. The size of the circle corresponds to the number of reported ZIKV sequences for that state, indicating areas with higher or lower viral activity. The lines represent the estimated spread routes for the virus, generated by Nextstrain. **A.** Spread map for all of Colombia. **B.** Spread map for Col01. **C.** Spread map for Col02. **D.** Spread map for Col03. The Spread maps were generated using NextStrain platform [58].
(TIF)

S1 File External contamination control amplification protocol [67, 68]. (PDF)
