# Supplementary material for: Genomic variability in Zika virus in GBS cases in Colombia
Source: PLoS One. 2024 Nov 19;19(11):e0313545. doi: 10.1371/journal.pone.0313545 (PMC11575819; doi:10.1371/journal.pone.0313545)
Supplement: S1 Table — (PDF) [file pone.0313545.s001.pdf]

**S1 Table.** Metadata of patients included in the study, sequencing method and NCBI accession.

| Patient | Date | City | Sample source | Sample type | Group | SRA accession | Genebank accession | Sequencing method | Age | Sex | Days from initial symptoms to sample | Disease features | CT |
|---|---|---|---|---|---|---|---|---|---|---|---|---|---|
| 88-07 | 2016-02-16 | Neiva | Urine | Clinical | GBS | SRR26779681 | PP431249 | Nanopore | 72 | male | 29 | Muscle pain, diarrhea, muscle weakness, lower limb paralysis, paresthesia | 27 |
| 88-03 | 2016-02-16 | Neiva | Urine | Clinical | GBS | SRR26779689 | PP431248 | Nanopore | 57 | female | 15 | Fever, headache, astenia, vomit, rash, conjunctivitis, facial palsy, muscle weakness, lower limb paralysis, paresthesia | 30 |
| 2-37 | 2016-06-02 | Cali | Urine | Clinical | GBS | SRR26779711 | PP431247 | Nanopore | 20 | female | 20 | Fever, headache, astenia, nausea, vomit, rash, altered mental status, muscle weakness, upper limb paralysis, lower limb paralysis, loss of sensation, paresthesia | 34 |
| 2-33 | 2016-05-11 | Cali | Urine | Clinical | GBS | SRR26779698 | PP431246 | Nanopore | 55 | male | 16 | Fever, joint pain, joint swelling, muscle pain, astenia, conjunctivitis, muscle weakness, lower limb paralysis | 32 |
| 2-31 | 2016-05-10 | Cali | Urine | Clinical | GBS | SRR26779712 | PP431245 | Nanopore | 44 | female | 12 | Joint pain, astenia, rash, conjunctivitis, muscle weakness | 32 |
| 2-28 | 2016-04-28 | Cali | Urine | Clinical | GBS | SRR26779707 | PP431244 | Nanopore | 26 | male | 10 | Fever, headache, joint pain, muscle pain, astenia, nausea, vomit, rash, conjunctivitis, altered mental status, muscle weakness, muscle rigidity, ataxia, seizures | 30 |
| 2-27 | 2016-04-27 | Cali | Blood | Clinical | GBS | SRR26779699 | PP431243 | Nanopore | 27 | male | NA | Muscle weakness, disautonomia | 31 |
| | 2016-04-27 | Cali | Urine | Clinical | GBS | SRR26779680 | PP431242 | Nanopore | | | | | 26 |
| 2-25 | 2016-04-21 | Cali | Urine | Clinical | GBS | SRR26779679 | PP431241 | Nanopore | 58 | male | 60 | Fever, rash, Facial palsy, muscle weakness, upper limb paralysis, lower limb paralysis, loss of sensation, paresthesia, neuropathic pain | 32 |
| 2-21 | 2016-04-05 | Cali | Urine | Clinical | GBS | SRR26779701 | PP431240 | Nanopore | 67 | male | 12 | Rash, conjunctivitis, lower limb paralysis, paresthesia | 24 |
| 2-18 | 2016-04-05 | Cali | Urine | Clinical | GBS | SRR26779709 | PP431239 | Nanopore | 47 | female | 36 | Fever, headache, rash, muscle weakness, lower limb paralysis | 35 |
| 2-15 | 2016-04-01 | Cali | Urine | Clinical | GBS | SRR26779703 | PP431238 | Nanopore | NA | male | NA | NA | 29 |
| 2-14 | 2016-04-01 | Cali | Urine | Clinical | GBS | SRR26779700 | PP431237 | Nanopore | NA | female | NA | NA | 24 |
| 2-01 | 2016-03-09 | Cali | Urine | Clinical | GBS | SRR26779678 | PP431236 | Nanopore | 41 | male | 13 | Joint pain, muscle pain, astenia, rash, conjunctivitis, facial palsy, loss of sensation, paresthesia | 30 |
| 13777 | 2016-03-07 | Cali | Urine | Clinical | GBS | SRR26779677 | PP431220 | Nanopore | 26 | male | 8 | Fever, joint pain, muscle pain, rash, conjunctivitis, muscle weakness, paresthesia | 29 |
| 13738 | 2016-02-01 | Cali | Blood | Clinical | GBS | SRR26779697 | PP431214 | Nanopore | 44 | male | 43 | | 32 |

| | | | | | | | | | | | | | |
|---|---|---|---|---|---|---|---|---|---|---|---|---|---|
| | 2016-02-04 | Cali | Blood | Clinical | GBS | SRR26779696 | PP431215 | Nanopore | | | 26 | | 25 |
| | 2016-02-06 | Cali | Urine | Clinical | GBS | SRR26779695 | PP431216 | Nanopore | | | 28 | | 18 |
| | 2016-02-06 | Cali | VERO cell line | Culture supernatant | GBS | SRR26779690 | PP431217 | Nanopore | | | NA | Fever, Muscle weakness, paresthesia, cardiorespiratory arrest, progressive quadriparesis | 14 |
| | 2016-02-06 | Cali | MiniBrain organoids | MiniBrain supernatant | GBS | NA | PP431251 | Illumina | | | NA | | 22 |
| 14053 | 2016-07-07 | Cali | Urine | Clinical | Non-GBS | SRR26779683 | PP431235 | Nanopore | 29 | female | 6 | Malaise, rash, joint pain, headache, retroocular, pain | 20 |
| 14012 | 2016-06-13 | Cali | Urine | Clinical | Non-GBS | SRR26779684 | PP431234 | Nanopore | 45 | female | 6 | Malaise, fever, rash, joint pain, conjunctivitis, headache, retroocular pain | 30 |
| 13995 | 2016-06-08 | Cali | Urine | Clinical | Non-GBS | SRR26779685 | PP431233 | Nanopore | 26 | female | 4 | Malaise, rash, joint pain, headache | 22 |
| 13960 | 2016-05-31 | Cali | Urine | Clinical | Non-GBS | SRR26779686 | PP431232 | Nanopore | 49 | female | 4 | Malaise, rash, joint pain, conjunctivitis, headache, retroocular pain | 27 |
| 13937 | 2016-05-18 | Cali | Blood | Clinical | Non-GBS | SRR26779705 | PP431231 | Nanopore | 69 | female | 2 | Malaise, fever, rash, joint pain, conjunctivitis, headache | 30 |
| 13930 | 2016-05-17 | Cali | Urine | Clinical | Non-GBS | SRR26779687 | PP431230 | Nanopore | 76 | female | 2 | Malaise, fever, rash, joint pain, conjunctivitis, headache, retroocular pain | 22 |
| 13843 | 2016-04-06 | Cali | Urine | Clinical | Non-GBS | SRR26779702 | PP431229 | Nanopore | 27 | female | 5 | Malaise, fever, rash, joint pain, conjunctivitis, headache, retroocular pain | 23 |
| 13841 | 2016-04-05 | Cali | Urine | Clinical | Non-GBS | SRR26779691 | PP431228 | Nanopore | 38 | female | 3 | Malaise, fever, rash, joint pain, headache | 31 |
| 13833 | 2016-04-01 | Cali | Urine | Clinical | Non-GBS | SRR26779682 | PP431227 | Nanopore | 22 | female | 10 | Malaise, fever, rash, joint pain, headache, retroocular pain | 25 |
| 13831 | 2016-03-31 | Cali | Urine | Clinical | Non-GBS | SRR26779692 | PP431226 | Nanopore | 78 | female | 3 | Malaise, fever, rash, joint pain, conjunctivitis | 25 |
| 13828 | 2016-03-31 | Cali | Urine | Clinical | Non-GBS | SRR26779693 | PP431225 | Nanopore | 47 | male | 4 | Malaise, fever, rash, joint pain, conjunctivitis, headache | 22 |
| 13823 | 2016-03-30 | Cali | Urine | Clinical | Non-GBS | SRR26779708 | PP431224 | Nanopore | 55 | male | 5 | Malaise, fever, rash, joint pain, conjunctivitis, headache, retroocular pain | 24 |
| 13820 | 2016-03-30 | Cali | Blood | Clinical | Non-GBS | SRR26779710 | PP431223 | Nanopore | 49 | male | 3 | Malaise, fever, rash. | 29 |
| 13785 | 2016-03-01 | Cali | C6/36 HT Cell line | Culture supernatant | Non-GBS | SRR26779694 | PP431222 | Nanopore | unknown (adult) | female | Unknown | Fever | 9 |
| | 03/01/16 | Cali | MiniBrain organoids | MiniBrain supernatant | Non-GBS | NA | PP431250 | Illumina | | | | | 20 |
| 13779 | 2016-03-09 | Cali | Urine | Clinical | Non-GBS | SRR26779688 | PP431221 | Nanopore | 30 | female | 3 | Malaise, fever, rash, joint pain, conjunctivitis, headache | 26 |
| 13754 | 2016-02-17 | Cali | Urine | Clinical | Non-GBS | SRR26779706 | PP431219 | Nanopore | unknown (adult) | male | Unknown | NA | 28 |
| 13740 | 2016-02-09 | Cali | Urine | Clinical | Non-GBS | SRR26779704 | PP431218 | Nanopore | 45 | male | 5 | Malaise, fever, rash, joint pain, conjunctivitis, headache | 30 |
