# Supplementary material for: Genomic variability in Zika virus in GBS cases in Colombia
Source: PLoS One. 2024 Nov 19;19(11):e0313545. doi: 10.1371/journal.pone.0313545 (PMC11575819; doi:10.1371/journal.pone.0313545)
Supplement: S2 Table — (PDF) [file pone.0313545.s002.pdf]

S2 Table. Primers and probes used for ZIKV detection by RT-qPCR.

Primers/Probe	Gene position	Sequence (5' -> 3')	Target	Reference
ZIKV 1086	1086–1102*	CCGCTGCCCAACAAG	E	40
ZIKV 1162c	1162–1139*	CCACTAACGTTCTTTTGCAGACAT		
ZIKV 1107-FAM	1107–1137*	AGCCTACCTTGACAAGCAGTCAGACACTCAA		
Zika 4481_F	4434–4453**	CTGTGGCATGAACCCAATAG	NS2B	41
Zika 4552c_R	4524–4505**	ATCCCATAGAGCACCACTCC		
Zika 4507c-FAM	4479–4460**	CCACGCTCCAGCTGCAAAGG		
RP-F	28-46***	AGATTTGGACCTGCGAGCG	RPP30	42
RP-R	73-92***	GAGCGGCTGTCTCCACAAGT		
RP-P	49-71***	TTCTGACCTGAAGGCTCTGCGCG		

* Position relative to the MR-766 strain of Zika virus (GenBank accession number AY632535).
** Position relative to the 2007 Yap strain of Zika virus (GenBank accession number EU545988).
*** Position on the accession of GenBank NM_001104546.2.
