# Supplementary material for: Genomic variability in Zika virus in GBS cases in Colombia
Source: PLoS One. 2024 Nov 19;19(11):e0313545. doi: 10.1371/journal.pone.0313545 (PMC11575819; doi:10.1371/journal.pone.0313545)
Supplement: S3 Table — (PDF) [file pone.0313545.s003.pdf]

S3 Table. ZIKV primers scheme.

Name	Sequence	Primer pool	Reference
ZIKA_400_1_LEFT	GACAGTTCGAGTTTGAAGCGAAAG	1	Quick et al. 2017
ZIKA_400_1_RIGHT	AGTATGCACTCCCACGTCTAGT	1	Quick et al. 2017
ZIKA_400_2_LEFT	AAGAAAGATCTGGCTGCCATGC	2	Quick et al. 2017
ZIKA_400_2_RIGHT	TGATTCCAACCAGGTTTGCGAC	2	Quick et al. 2017
ZIKA_400_3_LEFT	AGATGACGTCGATTGTTGGTGC	1	Quick et al. 2017
ZIKA_400_3_RIGHT	TACGGTGACACAACCTCCATGT	1	Quick et al. 2017
ZIKA_400_4_LEFT	TCAGGTGCGAGAGAGAGA	2	Quick et al. 2017
ZIKA_400_4_RIGHT	GGAGCCATGAACTGACAGCATT	2	Quick et al. 2017
ZIKA_400_5_LEFT	AGAACGTTAGTGGACAGAGGCT	1	Quick et al. 2017
ZIKA_400_5_RIGHT	TGTGCGTCCTTGAACTCTACCA	1	Quick et al. 2017
ZIKA_400_6_LEFT	TTGATTGTGAACCGAGGACAGG	2	Quick et al. 2017
ZIKA_400_6_RIGHT	CCATCTGTCCCTGCGTGTA	2	Quick et al. 2017
ZIKA_400_7_LEFT	TGAAGGGCGTGTCATACTCCTT	1	Quick et al. 2017
ZIKA_400_7_RIGHT	CGCCTCCAACTGATCCAAAGTC	1	Quick et al. 2017
ZIKA_400_8_LEFT	GGGAGAAGAAGATCACCCACCA	2	Quick et al. 2017
ZIKA_400_8_RIGHT	TTGACTGCTGCTGCCAATCTAC	2	Quick et al. 2017
ZIKA_400_9_LEFT	GCCTTAGGGGGAGTGTTGATCT	1	Quick et al. 2017
ZIKA_400_9_RIGHT	GAGTGGGCATTCCTTCAGTGTG	1	Quick et al. 2017
ZIKA_400_10_LEFT	ACGGTCGTTGTGGGATCTGTAA	2	Quick et al. 2017
ZIKA_400_10_RIGHT	GTGGGACTTTGGCCATTCACAT	2	Quick et al. 2017
ZIKA_400_11_LEFT	CAGCCGTTATTGGAACAGCTGT	1	Quick et al. 2017
ZIKA_400_11_RIGHT	CCTGGGCCTTATCTCCATTCCA	1	Quick et al. 2017
ZIKA_400_12_LEFT	CACTAAGGTCCACGTGGAGGAA	2	Quick et al. 2017
ZIKA_400_12_RIGHT	TATCAGCCAGATGAGCTACA	2	Quick et al. 2017
ZIKA_400_13_LEFT	TGGCAGTGCTGGTAGCTATGAT	1	Quick et al. 2017
ZIKA_400_13_RIGHT	SPIRITGAGGAGCATAACCCC	1	Quick et al. 2017
ZIKA_400_14_LEFT	CAATGGTTTTGCTTTGGCCTGG	2	Quick et al. 2017
ZIKA_400_14_RIGHT	TTTCCCATGTGATGTCACCTGC	2	Quick et al. 2017
ZIKA_400_15_LEFT	CCCTAGCGAAGTACTCACAGCT	1	Quick et al. 2017
ZIKA_400_15_RIGHT	TACACTCCATCTGTGGTCTCCC	1	Quick et al. 2017
ZIKA_400_16_LEFT	GTGGCATGAACCCAATAGCCAT	2	Quick et al. 2017
ZIKA_400_16_RIGHT	GCTCCAATGTCCCCATCCTTTG	2	Quick et al. 2017
ZIKA_400_17_LEFT	GTGGTCCATGGAAGCTAGATGC	1	Quick et al. 2017
ZIKA_400_17_RIGHT	CCTCTAAGGGCCTCCTCCATTT	1	Quick et al. 2017
ZIKA_400_17_RIGHT_alt	TAAGGGCYTCCTCCATTTCAGC	1	Present study
ZIKA_400_17_LEFT_alt	AGAGCGAGGAACATCCAGACT	1	Present study
ZIKA_400_18_LEFT	CTGTTGAGTGCTTCGAGCCTTC	2	Quick et al. 2017
ZIKA_400_18_RIGHT	TGGTGAGTTGGAGTCCGGAAAT	2	Quick et al. 2017
ZIKA_400_18_RIGHT_alt	TCCACTTCGGTGTCCATAATTGG	2	Present study
ZIKA_400_18_LEFT_alt	CCTGAAATAGTCCGTGAAGCCA	2	Present study

ZIKA_400_19_LEFT	TATGGATGAGGCCCACTTCACA	1	Quick et al. 2017
ZIKA_400_19_RIGHT	GCCATCAAGTATGACCGGCTTT	1	Quick et al. 2017
ZIKA_400_19_RIGHT_alt	CCAGAATGACTCTCTCGCCATC	1	Present study
ZIKA_400_19_LEFT_alt2	ATCTTCATGACCGCCACGCC	1	Present study
ZIKA_400_20_LEFT	GGCTGGAAAACGGGTCATACAG	2	Quick et al. 2017
ZIKA_400_20_RIGHT	CCTTTGCTCCGTCCTAAGCTTG	2	Quick et al. 2017
ZIKA_400_20_RIGHT_alt	CCCTCAATGGCTGCTACTTTGT	2	Present study
ZIKA_400_20_LEFT_alt	GGGTCATACARCTCAGCAGAAA	2	Present study
ZIKA_400_21_LEFT	AGAGACTGACGAAGACCATGCA	1	Quick et al. 2017
ZIKA_400_21_RIGHT	CTCCAAAAGCCGCTCCTCTTTT	1	Quick et al. 2017
ZIKA_400_21_RIGHT_alt	CTCCAAAARCSGCTCCTCTTTT	1	Present study
ZIKA_400_22_LEFT	TGGGAGAGAGAGAGAGA	2	Quick et al. 2017
ZIKA_400_22_RIGHT	ATTCTGGCTGGCTCAATTTCCG	2	Quick et al. 2017
ZIKA_400_22_2_RIGHT_alt	GAGGACACATGCAATTCTGGCT	2	Present study
ZIKA_400_22_2_LEFT_alt	TGGACGCCAGAGTTTGTTCAGA	2	Present study
ZIKA_400_23_LEFT	CGTCTTGATGAGGAACAAGGGC	1	Quick et al. 2017
ZIKA_400_23_RIGHT	AAGTGGTCACTGCATGTTGGAC	1	Quick et al. 2017
ZIKA_400_23_LEFT_alt	GGGCTTTGGAATGGTGACTCTT	1	Present study
ZIKA_400_23_RIGHT_alt2	CCGCAGGTCAATGTCCATTGA	1	Present study
ZIKA_400_24_LEFT	TAATGGGAAGGAGAGAGGAGGGGGGG	2	Quick et al. 2017
ZIKA_400_24_RIGHT	TCTCCACTTGGGGGTCAATTGT	2	Quick et al. 2017
ZIKA_400_25_LEFT	CCCTGACCTAGTGTGGCCAT	1	Quick et al. 2017
ZIKA_400_25_RIGHT	CCTTCCATTTCCCAGGGT	1	Quick et al. 2017
ZIKA_400_26_LEFT	ACTGGAACTCCTCTACAGCCAC	2	Quick et al. 2017
ZIKA_400_26_RIGHT	ACCAGGGCCTCCTTTTGTGTAT	2	Quick et al. 2017
ZIKA_400_27_LEFT	AGTGCAAAGCTGAGATGGTTGG	1	Quick et al. 2017
ZIKA_400_27_RIGHT	ATGTGTAGAGTTGCGGGAGAGT	1	Quick et al. 2017
ZIKA_400_28_LEFT	GGTGGGGGATTGGCTTGAAAAA	2	Quick et al. 2017
ZIKA_400_28_RIGHT	GGGCCTCATAGCTTCCATGGTA	2	Quick et al. 2017
ZIKA_400_29_LEFT	AGGATGTGAATCTCGGCTCTGG	1	Quick et al. 2017
ZIKA_400_29_RIGHT	ATGCTGCATTGCTACGAACCTT	1	Quick et al. 2017
ZIKA_400_30_LEFT	AAAAGTGGACACTAGGGTGCCA	2	Quick et al. 2017
ZIKA_400_30_RIGHT	TAATCCCAGCCCTTCAACACCA	2	Quick et al. 2017
ZIKA_400_31_LEFT	ACAAGGGGAATTTGGAAAGGCC	1	Quick et al. 2017
ZIKA_400_31_RIGHT	CGTAAGTGACAACTTGTCCGCT	1	Quick et al. 2017
ZIKA_400_32_LEFT	AAATGGAAAAAAAGGGCACAGGGC	2	Quick et al. 2017
ZIKA_400_32_RIGHT	TGTCCCATCCAGTTGAGGGTTT	2	Quick et al. 2017
ZIKA_400_33_LEFT	CAAACGAATGGCAGTCAGTGGA	1	Quick et al. 2017
ZIKA_400_33_RIGHT	ATCCACACTCTGTTCCACACCA	1	Quick et al. 2017
ZIKA_400_34_LEFT	ATTTCCACAGAAGGGACCTCCG	2	Quick et al. 2017
ZIKA_400_34_RIGHT	TGACTAGCAGGCCTGACAACAT	2	Quick et al. 2017
ZIKA_400_35_LEFT	ACCACCTGGGCTGAGAACATTA	1	Quick et al. 2017
ZIKA_400_35_RIGHT	ACCACTAGTCCCTCTTCTGGAG	1	Quick et al. 2017
