# Supplementary material for: Genomic variability in Zika virus in GBS cases in Colombia
Source: PLoS One. 2024 Nov 19;19(11):e0313545. doi: 10.1371/journal.pone.0313545 (PMC11575819; doi:10.1371/journal.pone.0313545)
Supplement: S4 Table — (PDF) [file pone.0313545.s004.pdf]

S4 Table. Haplotypes list.

Node Label	Matching sequences
1	KU820897.5<br>KX087102.2<br>MF574569.1<br>MF574574.1<br>MF574577.1<br>MF574552.1<br>MF574581.1<br>MF574588.1<br>MF574580.1<br>MF574582.1<br>MF574584.1<br>MF574586.1<br>MF574553.1<br>MF574583.1
2	MF574561.1
3	MF574573.1
4	MF574556.1
5	MF574570.1
6	MF574571.1
7	MF574562.1
8	MF574566.1
9	MF574558.1
10	MF574563.1<br>MF574564.1
11	MF574555.1
12	MF574568.1
13	MF574560.1
14	MF574576.1<br>MF574567.1<br>MF574575.1
15	MF574559.1
16	MF574572.1
17	KX247646.1
18	MF574565.1
19	MF574554.1
20	MF574557.1
21	MF574585.1
22	MF574587.1
23	KY989971.1
24	KY317940.1

25	KY317938.1
26	KY785466.1
27	MK049245.1
28	13828_O
29	MK049251.1
30	88_07_O
31	KY785469.1
32	MK049250.1
33	2_27_O
34	13823_O
35	2_15_O
36	13785_C636
37	OP898542.1
38	MH544701.2
39	OP898541.1
40	13937_S
41	13779_O<br>13740_O
42	13754_O
43	MK049247.1
44	2_25_O
45	KY317939.1
46	KY317936.1
47	88_03_O
48	13831_O
49	MH179341.1
50	2_28_O
51	13833_O
52	2_31_O
53	2_14_O
54	13843_O
55	13995_O
56	14053_O
57	KY317937.1
58	KX548902.1
59	MK049246.1
60	2_37_O
61	2_18_O
62	2_01_O
63	2_21_O
64	13738_O
65	2_33_O
66	13960_O

67	MK049248.1
68	MK049249.1
69	13841_O
70	13820_S
71	13777_O
72	13930_O
73	14012_O
74	MF574579.1
75	MF574578.1
