# Supplementary figures and images for: Genomic variability in Zika virus in GBS cases in Colombia
Source: PLoS One. 2024 Nov 19;19(11):e0313545. doi: 10.1371/journal.pone.0313545 (PMC11575819; doi:10.1371/journal.pone.0313545)

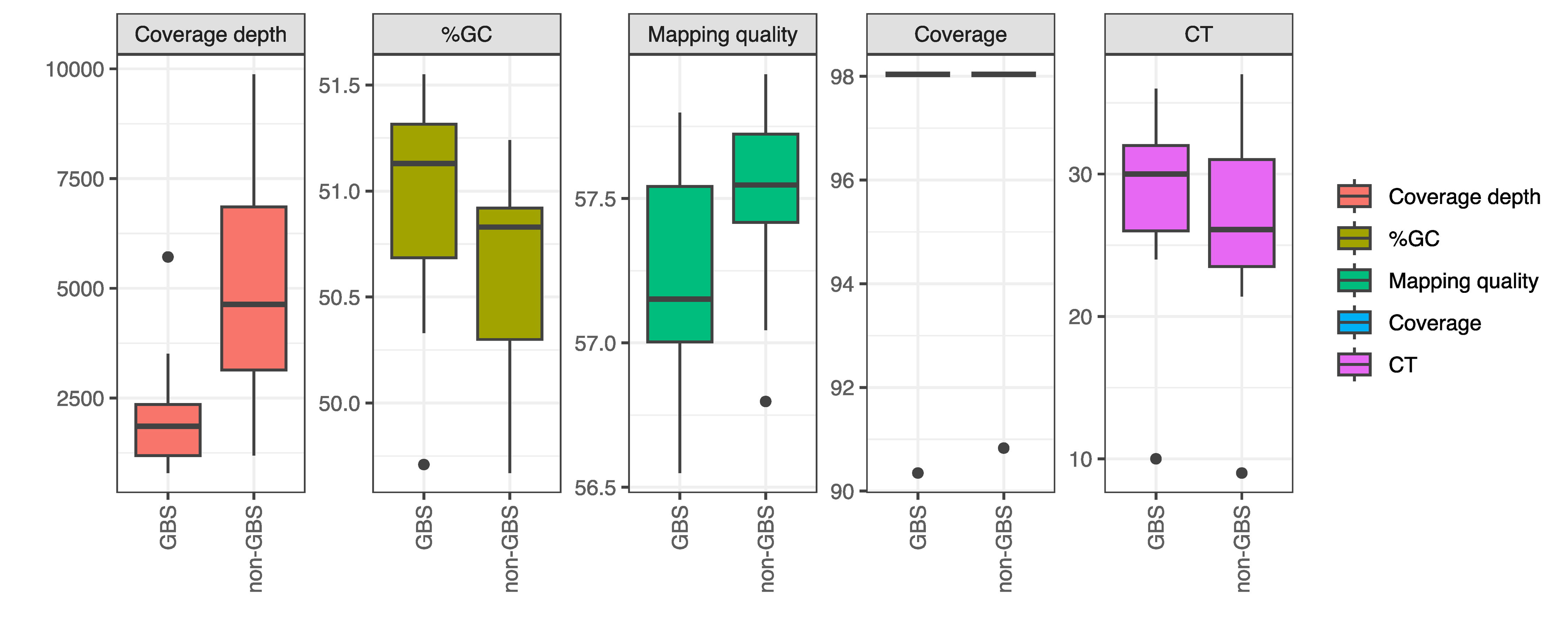

Supplement: S1 Fig — Descriptive of the assembled genomes of depth coverage mean, %GC, mapping quality, coverage, and cycle threshold (CT) of sequenced ZIKV genomes for the GBS and non-GBS groups. (TIF) [file pone.0313545.s005.tif]

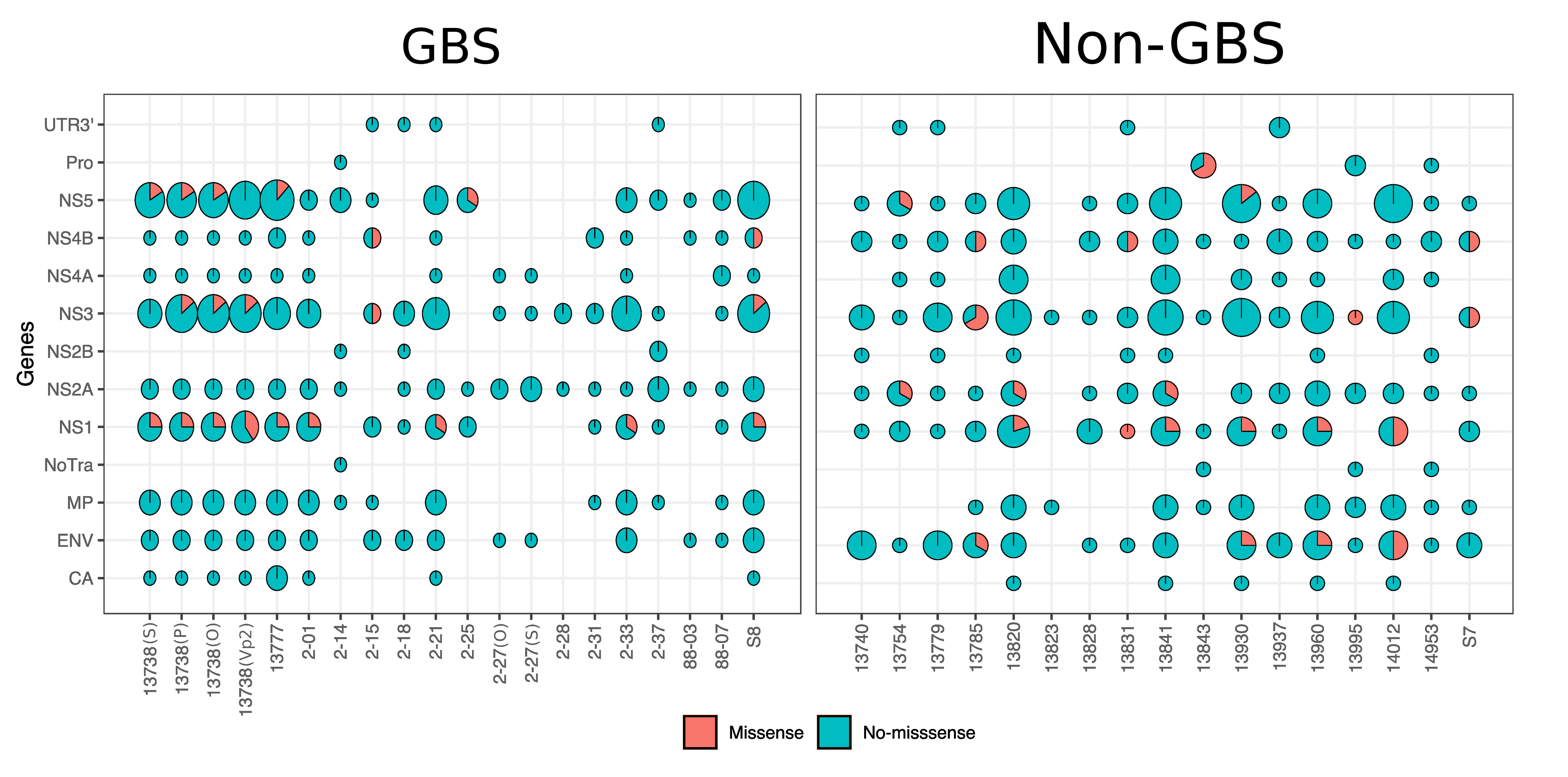

Supplement: S2 Fig — The size of the circles is proportional to the frequency of mutations with respect to the reference genome KJ776791.2. The cyan color indicates non-missense mutations, and the red color indicates missense mutations. (TIF) [file pone.0313545.s006.tif]

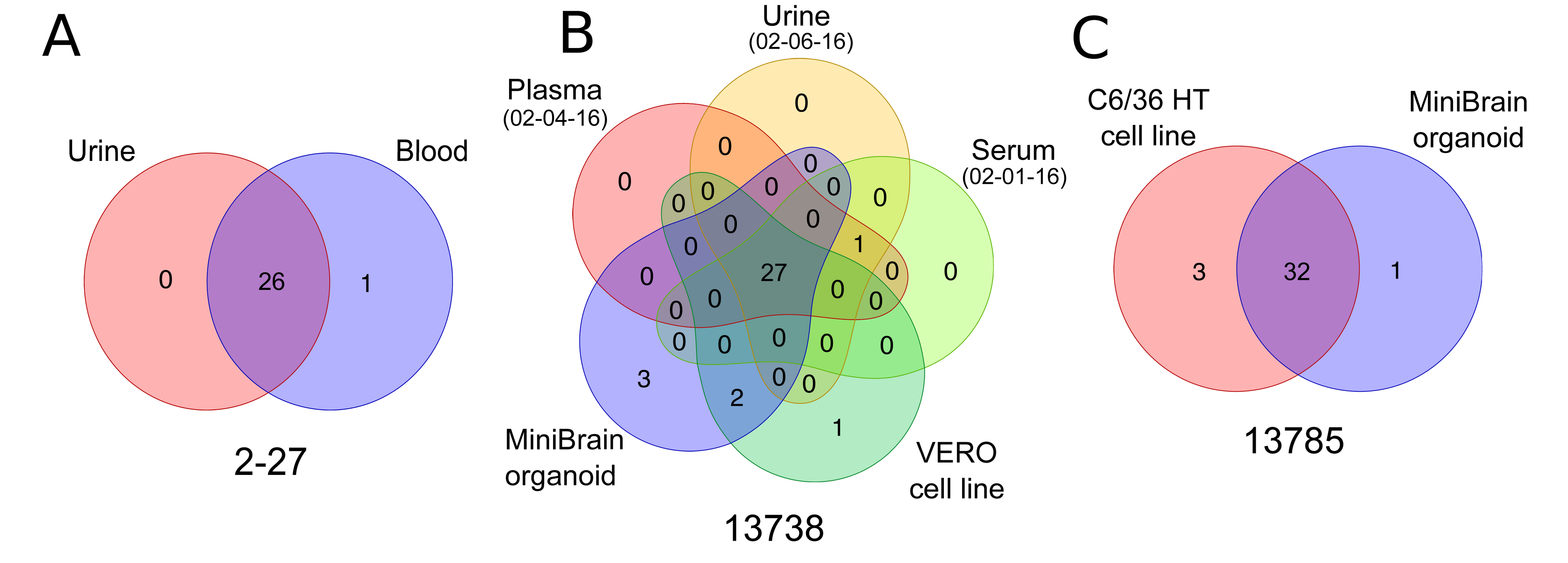

Supplement: S3 Fig — A. Comparison of ZIKV sequences of urine and blood samples from ZIKV-GBS 2–27 obtained by Nanopore. B. Comparison of ZIKV sequences obtained from blood (plasma and serum), urine, cell culture in VERO (Vp2) sequenced by Nanopore, and MiniBrains organoids sequenced by Illumina from ZIKV-GBS 13738. C. Comparison of ZIKV sequences from ZIKV-non-GBS 13785 that were cultured in C6/36 cells and sequenced by Nanopore or cultured in MiniBrains and sequenced by Illumina. All mutations are compared to the reference genome KJ776791.2. (TIF) [file pone.0313545.s007.tif]

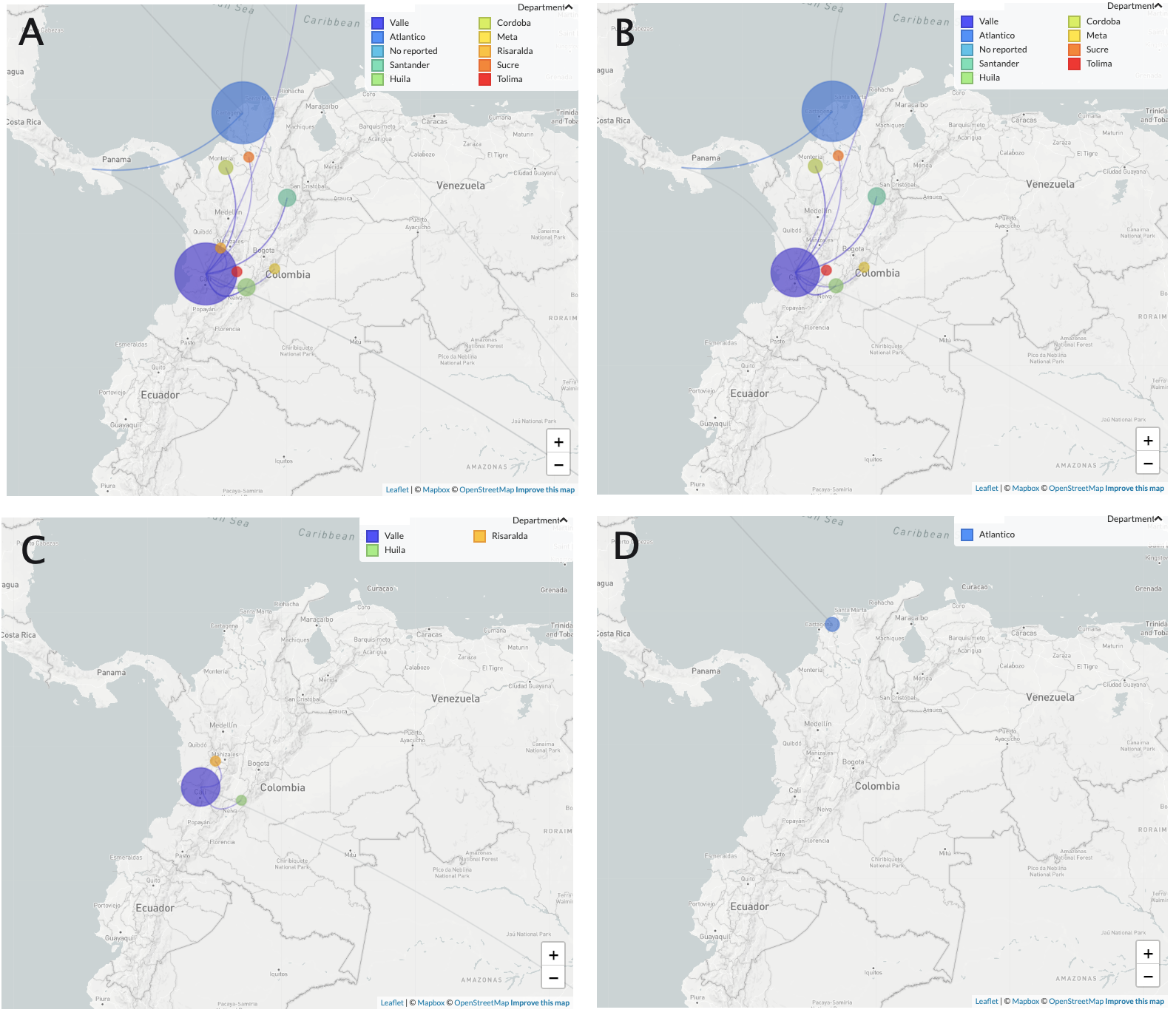

Supplement: S4 Fig — Geographic spread of the Zika virus (ZIKV) in Colombia during the 2016 outbreak. Each circle represents a state within Colombia where ZIKV sequences have been reported. The size of the circle corresponds to the number of reported ZIKV sequences for that state, indicating areas with higher or lower viral activity. The lines represent the estimated spread routes for the virus, generated by Nextstrain. A. Spread map for all of Colombia. B. Spread map for Col01. C. Spread map for Col02. D. Spread map for Col03. The Spread maps were generated using NextStrain platform [58]. (TIF) [file pone.0313545.s008.tif]
