# Supplementary material for: Genomic variability in Zika virus in GBS cases in Colombia
Source: PLoS One. 2024 Nov 19;19(11):e0313545. doi: 10.1371/journal.pone.0313545 (PMC11575819; doi:10.1371/journal.pone.0313545)
Supplement: S1 File — (PDF) [file pone.0313545.s009.pdf]

S1 File. External contamination control amplification protocol.

Amplification of the V3-V4 hypervariable region of the 16S rRNA gene was performed with primers 341F (5'-CTAYGGGRBGCASCAG-3) and 806R (5'-GGACTACNNGGGTATCTAAT-3') (60). For the 18S rRNA gene, the primers G3F1 (5' –GCCAGCAGCCGCGGTAATTC-3) and G3R1 (5' –ACATTCTTGGCAAATGCTTTCGCAG-3) were used (61). For the PCR mix, the following were used: 1X One Taq Standard Reaction Buffer; 200 µM dNTPs, 0.20 µM of each primer, 0.80 µg/µL of BSA, 0.625 U of Taq DNA polymerase, and between 2-5 uL of purified DNA, in a final volume of 25uL. For the 16s rRNA gene, an initial denaturation of 95ºC for 2 min, 38 cycles of 95ºC for 30 sec, 50ºC for 30 sec, and 68ºC for 30 sec, and a final extension of 68ºC for 5 min, were performed. For the 18S rRNA gene, an initial denaturation of 95ºC for 3 min was performed, followed by 25 cycles of 95ºC for 1 min, 60ºC for 1 min, and 72ºC for 30 sec, and a final extension of 72ºC for 4 min. Polymerase chain reaction (PCR) was performed on a Biorad CFX96 C1000 thermal cycler.
